# Empowering a Methanol-Dependent *Escherichia coli* via Adaptive Evolution Using a High-Throughput Microbial Microdroplet Culture System

**DOI:** 10.3389/fbioe.2020.00570

**Published:** 2020-07-09

**Authors:** Jia Wang, Xingjin Jian, Xin-Hui Xing, Chong Zhang, Qiang Fei

**Affiliations:** ^1^School of Chemical Engineering and Technology, Xi’an Jiaotong University, Xi’an, China; ^2^Department of Chemical Engineering, Tsinghua University, Beijing, China; ^3^Key Laboratory for Industrial Biocatalysis of the Ministry of Education, Tsinghua University, Beijing, China; ^4^Shaanxi Key Laboratory of Energy Chemical Process Intensification, Xi’an Jiaotong University, Xi’an, China

**Keywords:** microbial microdroplet culture, adaptive evolution, methanol-dependent *Escherichia coli*, co-substrate reduction, whole-genome sequencing, automatic high-throughput

## Abstract

Recently, a methanol-essential *Escherichia coli* was constructed; this strain is highly dependent on a supply of gluconate as a co-substrate for growth. Adaptive laboratory evolution is commonly applied to obtain mutants with specific phenotypes under certain selected pressure. However, conventional adaptive evolution approaches are not only laborious and time consuming, but they also come with lower throughput and inefficiency. In order to empower the aforementioned *E. coli* with reduced gluconate usage and enhanced growth rate, an irrational strategy based on a microbial microdroplet culture (MMC) platform was developed in this study. Given the automatic high-throughput selection of the MMC, a three-stage regime of an adaptive evolution experiment via gradually decreasing the availability of gluconate during the cultivation was performed for 50 days continuously in order to obtain the mutations. Finally, a candidate mutant was obtained with a 3-fold faster growth rate, a 43% shorter lag phase, and 40% less gluconate usage compared with the starting strain. Moreover, the gene mutations of *gntU, idnT, edd*, and *pckA* were identified by analyzing the whole-genome sequencing of mutants, which are strongly associated with the efficiency of gluconate uptake and cell growth. In conclusion, we have successfully demonstrated the feasibility of using MMC platform to empower the target strain with specific requirements in a manner of labor, time efficiency, and directed evolution.

## Introduction

Due to the steady increase in both the global population and the demand for food (Ganesh, 2014), it is urgent to explore more available substrates with which to replace limited editable feedstocks used as carbon sources for biomanufacturing (Wen-Liang et al., 2016; Hu et al., 2017; Wang et al., 2017; Fei et al., 2018, 2020; Cui et al., 2018a). Methanol as an essential key platform compound can be obtained from various sources on a megaton scale (Schrader et al., 2009; Pellegrini et al., 2011; Bertau et al., 2014; Haynes and Gonzalez, 2014; Hu and Lidstrom, 2014; Bennett et al., 2017). Nowadays, methanol is applied as a substrate in many bioconversion processes due to the application of advanced genetic engineering under rational and irrational designs (Schrader et al., 2009; Bennett et al., 2017).

Although methylotrophs can naturally consume methanol as a sole carbon source for growth and production, few methylotrophs can be used as model strains for far-ranging synthetic research studies (Whitaker et al., 2015; Nguyen et al., 2016; Zhang et al., 2017). Therefore, genetic engineering of *Escherichia coli* capable of assimilating methanol has attracted considerable interest in terms of biosynthesis and fermentation applications (Cocks et al., 1974; Müller et al., 2015; Whitaker et al., 2015; Bennett et al., 2018; Gonzalez et al., 2018). Recently, a methanol-dependent *E. coli* was developed by heterotrophically expressing ribulose monophosphate cycle (RuMP) with *mdh* (methanol dehydrogenase), *hps* (3-hexulose-6-phosphate synthase), and *phi* (6-phospho-3-hexuloisomerase) genes, which enabled *E. coli* to utilize methanol directly as a carbon source (Meyer et al., 2018). Nevertheless, it is worth noting that the growth of the aforementioned *E. coli* strongly relies on the supply of gluconate as a co-substrate for growth, which drastically limits the versatile of this strain in commercialization. This consequently reduces the usage of substrate during the culture of this methanol-dependent *E. coli*, and this is thus one of the key puzzles leading to its applications in the future.

Given the limited understanding of the methylotrophic metabolic performance in heterogeneous hosts, optimizing the growth and substrate utilization rates based on rational designs is challenging. Instead, irrational adaptive evolution makes phenotypic changes clearly related to specific growth environments directing to trait selection (Hardison, 2003; Cui et al., 2018b). In addition, with the benefit of new technologies including transcriptome sequencing (Schena et al., 1995) and whole-genome resequencing (WGS; Sniegowski et al., 1997; Lenski et al., 1998; Cooper and Lenski, 2000), phenotype–genotype correlations can be easily obtained. In the last decades, the adaptive evolution strategy has been employed to obtain the targeted mutations of different strains (Dragosits and Mattanovich, 2013; Hong and Nielsen, 2013; Wu et al., 2013; Xue et al., 2016), which led to important insights and experimental proof for evolutionary biology. Adaptive evolution approaches, however, are always hurdled by the requirements of increased fitness for identifying improved phenotypes or property, which causes massive parallel cultures with indispensable monitoring and control (Dettman et al., 2012; Andrew and Graham, 2013). Clearly, conventional cultivation techniques, i.e., shake flasks and well plates (Weuster-Botz et al., 2007), could not achieve their final goals efficiently due to the low data density (usually only end-point measurements), long time span, poor parallelism, and distribution monitoring (Hemmerich et al., 2017). Thus, the development of an automated, modularized microbial cell micro-cultivation system, particularly a system based on droplet microfluidics (Jakiela et al., 2013), has gained attention in microbiology for its high-throughput (Baraban et al., 2011; Ota et al., 2019), parallelized (Kaushik et al., 2017), and highly efficient adaptive laboratory evolution capability and decreased chances for human error during cultivation (Churski et al., 2012; Jian et al., 2019).

In this study, a microbial microdroplet culture (MMC) system (Jian et al., 2019) was employed to develop a novel strategy to both improve the cell growth of methanol-dependent *E. coli* and reduce the amount of co-substrates used during cultures. The cultivation performance of MMC has been validated first by comparing cell growth curves and methanol volatilization in MMC with that in conventional shake flasks and well plates. The MMC was investigated for adaptive evolution in a three-stage cultivation developed via gradually decreasing the availability of gluconate in the culture medium. Mutant strains with better growth rates and less gluconate usage obtained from MMC were selected for whole-genome sequencing to reveal the key genes associated with the mutations, which lays a foundation for further improving the methanol utilization capacity of the mutants.

## Materials and Methods

### Strains and Culture Medium

*Escherichia coli* MeSV2.2 used in this study was obtained from Prof. Julia Vorholt and maintained and cultured as described previously (Meyer et al., 2018). The seed was first cultured under 37°C with 200 rpm in shake flasks for 84 h and then grown on MMC at 50 droplets at for another 48 h at 37°C. In this study, 2% inoculation was used for our experiments and the passage time in MMC was set to 30 h. The carbon source used in the medium was gradually reduced to 500 mM methanol and 3 mM sodium gluconate based on the original 500 mM methanol and 5 mM sodium gluconate. All chemicals used were of analytical grade.

### Operational Procedures for the MMC

Microbial microdroplet culture is set up by using pumps and valves, reagents bottles, a droplet manipulation fluidic chip, and a microbial cell cultivation tube as reported before (Jian et al., 2019). Based on the material characteristics of the chips and cultivation tube, the UV irradiation for 30 min was used for sterilization. The droplet volume of 2.00 μL with a total number of 50 was set up in this study. For the status of growth and instrument operation, the OD_600_ value and the number of droplets when the droplets pass through the detection site were measured. In our experiments, the droplet detection interval was 27 min. More detailed information regarding the MMC setup system can be found in the previous article (Jian et al., 2019).

### Cultivation and Passage of *E. coli* MeSV2.2

To determine the optimal evolutionary strains, the starting strains of all experiments were from the best strain of the previous stage, which were cultivated in a shake flask for 72 h and transferred to MMC whereupon they were inoculated with fresh medium at a concentration of 2%. Since the growth cycle of the strain MeSV2.2 was longer, the *E. coli* MeSV2.2 was cultivated in shake flask for 5 h before seeding into MMC. In our study, 50 droplets were generated in every adaptive evolution experiment, and the concentration of inoculum was set to 15% in each passaging experiment. The duration of each passaging experiment was 30 h, and the medium was then replaced with a fresh medium by progressive division and fusion. The growth curves of 50 droplets were displayed in real time based on the online cell density measurement results. When the curve showed a clear peak, the droplets were extracted to verify the evolution result in shake flasks. The evolutionary strain was selected with the highest growth rate in the shake flask verification experiment for the adaptive evolution of the next generation.

In the case of the droplets were extracted for verification, the calibration method of the measurement of OD_600_ in MMC and well plates was described in the previous article (Jian et al., 2019). In this study, the growth rate was calculated by using OD_600_ value of samples collected (or directly measured) during the logarithmic phase, which began at the end of lag phase (12 h). In order to give a correct comparison, the samples were measured from the same time period when the cultures were performed in MMC, shake flasks, and well plates. The growth rate was shown as mean ± standard deviation (*n* = 3 for shake flasks, *n* = 48 for well plates, *n* = 50 for MMC), and propagation of error was carried out according to the variance formula. Statistical analysis was performed in Microsoft Excel and *P*-values with statistical significance of *P* < 0.05 and *P* < 0.01 were obtained.

### Methanol Concentration Measurement

To prepare a calibration curve for methanol, the following standard solutions were prepared: 2471, 1977, 989, 494, 247, 124, and 0 mmol/L methanol standard solution. The methanol standard solution was transferred to the gas phase vial, and the methanol content was determined by gas chromatography (GC 2010, Shimadzu, Japan) based on the measurement described in a previous article (Whitaker et al., 2016).

### WGS and Mutational Analysis

Next generation sequencing (NGS) library preparations were constructed following the NEBNext^®^ Ultra^TM^ DNA Library Prep Kit for Illumina^®^ (Milse et al., 2014). Based on the manufacturer’s protocol, the libraries of multiple indexes were performed on an Illumina HiSeq instrument (Illumina, San Diego, CA, United States) with a 2 × 150 paired-end (PE) configuration. The map clean data was obtained by removing the adapter sequence, polymerase chain reaction (PCR) primers, the content of N bases more than 10%, and bases of a quality lower than 20. The sequence of starting strain was used to reference the genome. Genomic structural variation analysis was performed by Pindel (version 0.2.5b8) and CNVnator (version 0.3.1).

## Results and Discussion

### Characterizations of the Cultivation Performance in MMC

**FIGURE 1:**
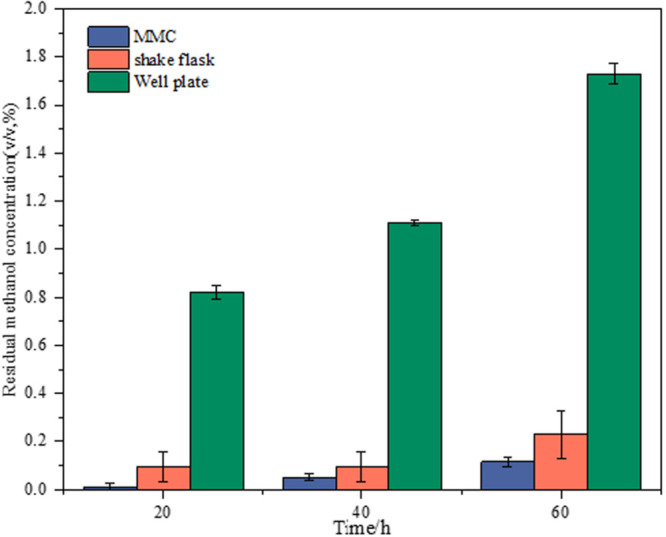
Comparison of volatilization-proof characterization of shake flasks, well plates, and MMC based on the menthol concentration left in the cell-free medium during cultivation.

The adaptive evolution experiments are always time-consuming, which requires good stability of maintaining substrate concentration during cultivation, especially for volatile substrates such as methanol. Therefore, the methanol concentration left in the cell-free medium during the cultivation was investigated for volatilization-proof characterization in the platform of MMC, shake flasks, and well plates. The starting samples of all experiments were from the same medium with 2% methanol, which were then individually transferred to 96-well plates, shake flasks, and MMC. The profiles of residual methanol concentration at different sampling times in various platforms are showed in Figure 1. Comparing with shake flasks and well plates, the MMC showed the lowest amount of methanol volatilization and the fluctuation of the volatilization amount presented minor changes with the prolongation of time. This finding could be explained by the fact that, as a compartmentalized cultivation system, carrier oil in MMC insulates droplets from the environment, thus preventing methanol in droplets from evaporating. It was worth noting that although the methanol volatilization in shake flasks was higher than MMC, it has obvious advantages over the well plate, which was not suitable for our purpose.

**FIGURE 2:**
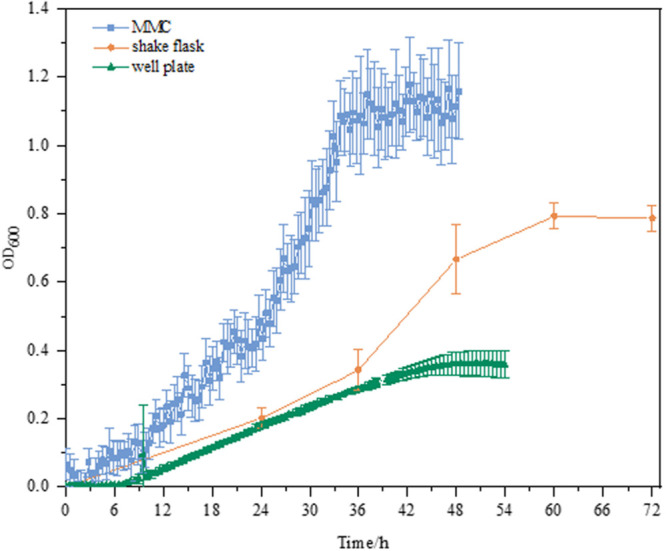
Growth curves of MeSV2.2 in shake flasks, well plates, and MMC. All experiments were finished when cell growth reached the stationary phase. All the growth curves are calibrated by plotting the calibration curve for OD_600_ value correlated to the absorbance at 600 nm measured by UV/VIS spectrophotometer for both well plates and MMC.

As shown in Figure 2, the culture performance using different culture platforms was in good agreement with our aforementioned findings (Figure 1). *E. coli* MeSV2.2 exhibited much better growth in MMC in terms of the OD and specific growth rate. Although the growth rates of three culture platforms were similar within 12 h, and significant differences between 12 and 36 h were obvious. Compared to the growth rate of 0.023 h^–1^ ± 0.00063 in shake flask and 0.0097 h^–1^ ± 0.00082 in the well plate, the highest growth rate of 0.032 h^–1^ ± 0.00345 was achieved in MMC. This may be because of the characteristics of MMC in terms of larger total surface area to volume ratio, higher transfer rate, the increased oxygen supported from the Teflon membrane tubing, as well as rapidly removing carbon dioxide trapped in the medium. Besides, the higher growth observed in MMC may be also due to the sufficient methanol kept in the medium during cultivation using MMC (no methanol was detected at the end of all experiments). Based on the above experimental result, MMC was finally validated as the most suitable platform to carry out the next adaptive evolution.

### Adaptive Evolution of *E. coli* MeSV2.2 in MMC

**FIGURE 3:**
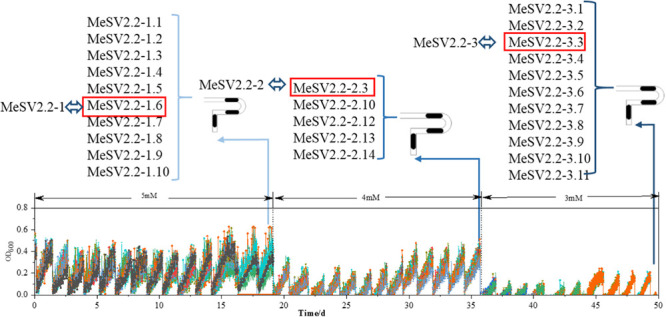
Results of the adaptive evolution of MeSV2.2 in MMC for 50 days in three different stages. The growth curves of MeSV2.2 by continuous sub-cultivation. In the course of the whole process, the highest points of growth curves in each sub-cultivation first fell and then rose overall, resulting in the valley and peak. In three-stage of adaptive evolution, the adaptively evolved strains were separately extracted from MMC, which were then named, as shown in Figure 3. The red boxes indicate strains selected for shake flask growth curve measurement and rename.

**FIGURE 4:**
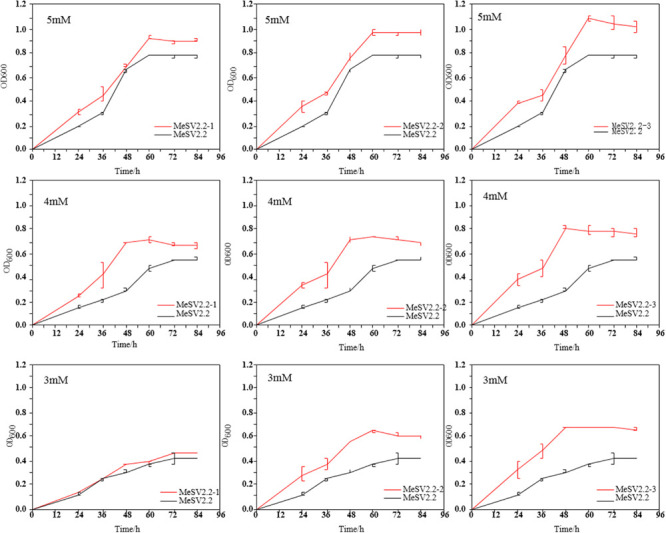
Growth curve of extracted evolved bacteria in shake flasks. Comparison of the growth curves of three selected strains (MeSV2.2-1, MeSV2.2-2, and MeSV2.2-3) in three different stages with the starting strain (MeSV2.2). The values of 5, 4, and 3 mM in this Figure correspond to the sodium gluconate of 5, 4, and 3 mM, which was used as co-substrates along with 500 mM methanol in these cultures.

In order to obtain promising mutants of methanol-dependent *E. coli* MeSV2.2 with better growth and less co-substrate usage, three stages of adaptive evolution were performed under the same culture conditions with adding different concentrations of sodium gluconate across all three stages. As shown in Figure 3, methanol of 500 mM along with 5 mM (in the 1st stage), 4 mM (in the 2nd stage), and 3 mM (in the 3rd stage) sodium gluconate were supplemented as a selection pressure for adaptive evolution. Each adaptation experiment was carried out in a time span from 14 to 20 days until a candidate mutant was obtained for the next stage. After a 50-day continuous adaptation cultivation, the MeSV2.2-1, MeSV2.2-2, and MeSV2.2-3 was selected from the 1st, 2nd, and 3rd stage, respectively, which were evaluated thoroughly in shake flask cultures for better observation as shown in Figure 4.

It was clear that an obvious increase in growth rate was achieved for all evolutionary mutants as compared to the starting strain (*E. coli* MeSV2.2) at different concentrations of sodium gluconate. As a cumulative result, the MeSV2.2-3 mutant showed a higher growth rate of 0.0184 h^–1^ in 5 mM sodium gluconate, 0.0166 h^–1^ in 4 mM sodium gluconate, and 0.0142 h^–1^ in 3 mM sodium gluconate, which corresponded with the 0.4X, 2X, and 3X faster growth rate of the starting strain under the same circumstances. A similar trend of enhancements could be also found in MeSV2.2-1 and MeSV2.2-2 in terms of the growth rate. As can be seen in Figure 4, MeSV2.2-3 exhibited the highest growth than the other two mutants obtained from the 1st or 2nd stage. It needs to be noted that MeSV2.2-3 also gave a 43% shorter lag phase when reducing sodium gluconate supply by 40%, indicating a promising mutant being less dependent on cosubstrate could be achieved using our MMC platform with irrational designs. To comprehensively understand the mutations at the genome level, the WGS of MeSV2.2-1, MeSV2.2-2, and MeSV2.2-3 were conducted and analyzed.

### WGS Analysis

**FIGURE 5:**
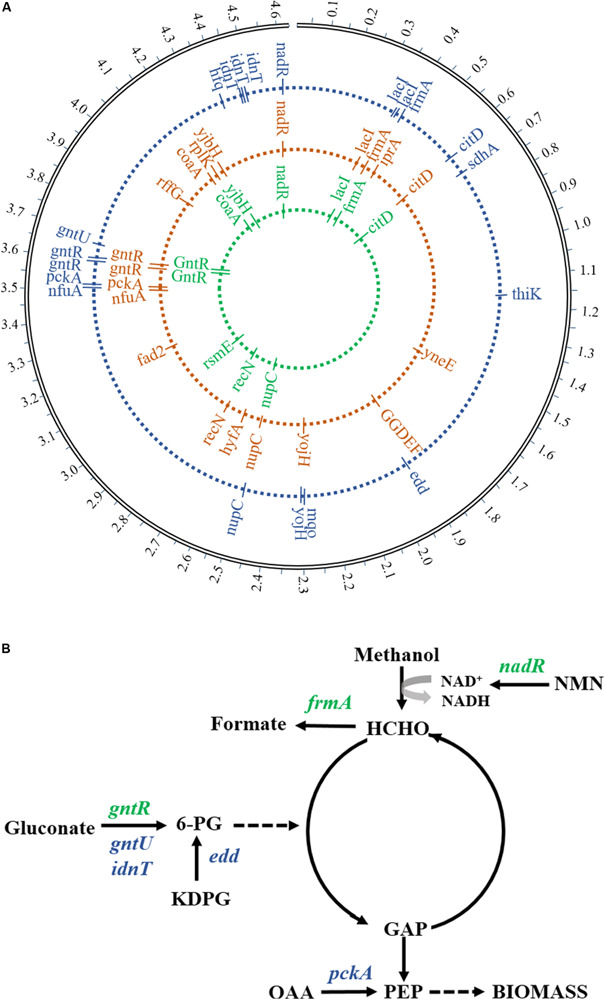
Mutated genes in adaptive evolution. **(A)** The locus map of mutations showed the location of mutated genes during adaptive evolution. From the inside to the outside, the mutant genes in the first stage, the mutant genes in the second stage, and the mutant genes in the third stage are respectively represented. **(B)** Major mutated genes involved in carbon metabolic flux are shown. Green indicates the mutant gene from the first evolution and blue indicates the mutant gene from the third evolution. Enzymes (genes): binding transcriptional repressor (*gntR*), glutathione-dependent formaldehyde dehydrogenase (*frmA*), DNA-binding transcriptional repressor/nicotinamide mononucleotide adenylyltransferase (*nadR*),low-affinity gluconate transporter (*gntU)*, Gnt-II system L-idonate transporter (*idnT*), phosphogluconate dehydratase (*edd*), and phosphoenolpyruvate carboxykinase (*pckA*). Metabolites: glucose 6-phosphate (6-PG), nicotinamide mononucleotide (KDPG), glyceraldehyde 3-phosphate (GAP), fructose 6-phosphate phosphoenolpyruvate (PEP), oxaloacetate (OAA), and nicotinamide mononucleotide (NMN).

The WGS was performed in order to find potential genotypes for the growth phenotype of evolutionary strains. By analyzing and comparing genome sequencing data, seven prominent gene mutations related to methanol metabolism and gluconate utilization were detected in mutants compared to the starting strain (Figure 5A), including DNA-binding transcriptional repressor (*gntR*), glutathione-dependent formaldehyde dehydrogenase (*frmA*), DNA-binding transcriptional repressor/nicotinamide mononucleotide adenylyltransferase (*nadR*), low-affinity gluconate transporter (*gntU)*, Gnt-II system L-idonate transporter (*idnT*), phosphogluconate dehydratase (*edd*), and phosphoenolpyruvate carboxykinase (*pckA*). The gene mutations of *gntR*, *frmA*, and *nadR* found from the 1st-stage mutant stain (MeSV2.2-1) were the same as in the previous results obtained from a one-stage adaptive evolution experiment carried out in flasks (Meyer et al., 2018). Moreover, four more mutation genes related to gluconate uptake were discovered in MeSV2.2-3 after three stages of the adaptive evolution via using the MMC (Figure 5B). This finding is consistent with what we observed in the culture of MeSV2.2-3, requiring less gluconate with better growth.

It has been reported that gluconate utilization is negatively regulated by GntR through the Gnt-I system, and that GntU—closely homologous to GntR (*gntR*)—can significantly inhibit gluconate uptake in *E. coli* (Faik and Kornberg, 1973; Tong et al., 1996). In our study, missense mutations in both *gntR* and *gntU* were observed and are likely to cause a loss of function rather than enhance, taking into account the improved efficiency of gluconate intake. Another mutation was observed in the gene *idnT* encoding a high-affinity gluconate transporter responsible for regulating the Gnt-II system with the transportation of L-idonate, which can be further catalyzed to form 6-phosphogluconate (Bausch et al., 1998). A non-frameshift insertion in exon regions of *idnT* is predicted to be the result of the increase in length of the coding region, causing improved transportation efficiency of L-idonate and finally resulting in enhanced gluconate uptake (less usage of gluconate). Surprisingly, the mutation of *edd*—responsible for the dehydration of 6-phospho-D-gluconate to 2-dehydro-3-deoxy-6-phospho-D-gluconate—was found, which has been previously knocked out in MeSV2.2 (Meyer et al., 2018). The frameshift insertion from the WGS analysis suggests more bases have been inserted, which resulted in the loss-of-function genes. The mutation of *edd* may be induced by the increased accumulation of 6-phospho-D-gluconate caused by the mutations of *gntR*, *gntU*, and *idnT*, providing an enhanced intake rate of gluconate. Because of the mutation of *edd*, the flux of 6-phospho-D-gluconate can be redirected to generate more GAP for pyruvate production.

Interestingly, a mutation was observed in Pck (*pckA*), which catalyzes oxaloacetate (OAA) into fructose 6-phosphate phosphoenolpyruvate (PEP). Hou et al. (1995) have reported that controlling the activity level of the ATP-dependent Pck is essential for a higher growth rate of *E. coli* cultured on succinate. Thus, the missense mutation of *pckA* in exon regions may act a global regulation of *pckA* expression, which gives interpretation to the faster growth rate of MeSV2.2-3. The mechanism of this finding is still required further investigation.

## Conclusion

In this study, MMC-based adaptive evolution was employed for improving cell growth of methanol-dependent *E. coli* with less co-substrate usage. A mutant of MeSV2.2-3 was finally obtained from a three-stage evolution experiment, which exhibited much better growth performance with 40% less sodium gluconate usage compared to the starting strain. For the first time, our WGS analysis revealed three mutation genes (*gntU*, *idnT*, and *edd*) related to the enhanced co-substrate-independent capability of MeSV2.2-3. Our results demonstrated that specific phenotypes could be achieved via the irrational design with the use of MMC, providing a powerful platform for automatic high-throughput adaptive evolution.

## Data Availability Statement

The raw datasets generated for this study can be found in the Bioproject archive, accession number PRJNA629683.

## Author Contributions

CZ and X-HX conceived the experiment and manuscript. CZ and JW designed the experiments. JW and XJ performed all the experiments. QF and CZ guided the experiments and analyzed the data. QF and JW wrote the manuscript. CZ and X-HX edited the manuscript. All authors read and approved the final version of the manuscript.

## Conflict of Interest

The authors declare that the research was conducted in the absence of any commercial or financial relationships that could be construed as a potential conflict of interest.

## Abbreviations

MMC: microbial microdroplet culture system
NGS: next-generation DNA sequencing
OD: optical density
RuMP: ribulose monophosphate cycle
WGS: whole-genome resequencing.
